# Dosimetry study of three-dimensional print template for 125I implantation therapy

**DOI:** 10.1186/s13014-021-01845-y

**Published:** 2021-06-24

**Authors:** Enli Chen, Yuwei Zhang, Hongtao Zhang, Chenfei Jia, Yansong Liang, Juan Wang

**Affiliations:** 1 Graduate School of Hebei Medical University, Shijiazhuang, Hebei China; 2 Department of Oncology, Hebei General Hospital, 348 West Heping Road, Shijiazhuang, 050051 Hebei China

**Keywords:** Brachytherapy, Iodine-125, 3D printing

## Abstract

**Background:**

^125^I seed implantation has been found to show good therapeutic effects on tumors. Recent studies showed that three-dimensional (3D) print template-assisted ^125^I seed implantation can optimize radiation dose distribution. This study aimed to compare the dose distribution differences in ^125^I seed implantation among 3D print noncoplanar template- (3DPNCT), 3D print coplanar template- (3DPCT) assisted implantation and traditional free-hand implantation.

**Methods:**

We systematically searched the PubMed, EMbase, Cochrane Library, Wan Fang Med Online, China National Knowledge Infrastructure (CNKI) from the earliest to November 2020 without time or language restrictions. And the references of primary literature were also searched. The outcome measures were dosimetry and operation time. This meta-analysis was carried out using Stata 12.0.

**Results:**

A total of 16 original articles were selected for inclusion. The differences of D90, D100, V90, and V100 values pre- and post-implantation with traditional free-hand implantation showed statistically significant (*p* < 0.05). The differences of D90, D100, V100, V150, V200, and D2cc of organs at risk (OAR) values pre- and post-implantation with 3D print template showed no statistically significant (*p* > 0.05). Compared with traditional free-hand implantation without any templates, 3D print template could improve postoperative D90 (Standard mean difference, SMD = 0.67, 95% confidence interval (CI) = 0.35 to 0.98, *p* < 0.001), D100 (SMD = 0.82, 95%CI = 0.40 to 1.23, *p* < 0.001), V90 (SMD = 1.48, 95%CI = 0.95 to 2.00, *p* < 0.001), V100 (SMD = 1.41, 95%CI = 0.96 to 1.86, *p* < 0.001), and reduce operation time (SMD = − 0.93, 95%CI = − 1.34 to − 0.51, *p* < 0.001). In three studies, both 3DPNCT and 3DPCT plans were designed for all patients. The prescribed dose and seed activity were same. Pooled analysis of D90, D100, V100, D2cc of OAR, number of seeds and number of needles showed no significant differences between 3DPNCT and 3DPCT groups (*p* > 0.05). However, in 3DPNCT group, V150 and V200 were increased (SMD = 0.35, 0.49; 95%CI = 0.04 to 0.67, 0.02 to 0.96; *p* = 0.028, 0.043); the number of through bone needles was reduced (SMD = − 1.03, 95%CI = − 1.43 to − 0.64, *p* < 0.001).

**Conclusions:**

Compared with traditional free-hand implantation, 3D print template-assisted ^125^I seeds implantation can optimize dose distribution and reduce the implantation time at the same time. Compared with 3D print coplanar template, 3D print noncoplanar template can increase the volume of high dose within tumor target and is more safer in the respect of puncture route.

**Supplementary Information:**

The online version contains supplementary material available at 10.1186/s13014-021-01845-y.

## Background

Recently, radioactive iodine—125 seeds (RIS) implantation has been widely applied to treat various malignant tumors and has achieved satisfactory therapeutic effects [1, 2]. RIS has the features of a minor trauma surgery, delivering a high local radiation dose to tumor targets and sharply dropping off at adjacent normal tissues. Nevertheless, up to date, there was still no standard procedures for 125I seed implantation for other tumors in the body except prostate cancer. It was challenging to effectively preplan for seed implantation resulted from patients’ body movement, organ movement, and bone structure interference. RIS implantation just relied on individual clinical experience and puncture techniques. Seed location and dose distribution were not the same as the preplan, which could lead to complications of operation and local recurrence of tumor.

In term of high-dose-rate (HDR) brachytherapy, Martinez developed an afterloading applicator that consisted of an template with a predrilled holes which were used as guides for trocars in 1984. And so, trocars could be inserted through the holes and produced optimal dose coverage of the tumor volume, which could reduce the degree of technical difficulty and improve the dose-rate distributions [3]. Aristei designed an 3D template which was confirmed to be a quick, easy, reliable and time-saving method to localize the volume of tumor target for HDR brachytherapy in breast cancer patients [4]. Mahantshetty included 113 patients with gynecologic cancers treated with template-based HDR interstitial brachytherapy boost, which resulted in a satisfactory clinical outcomes without any severe toxicities [5]. Coincidentally, doctors in China tried to implant RIS with the template which was designed individually by 3D printer. The use of 3D print templates including 3D print noncoplanar template (3DPNCT) and 3D print coplanar template (3DPCT) made it more precise to implant RIS, with a highly consistent dose distribution of target volume. Many studies showed that 3D print template-assisted RIS implantation could not only reduce the dosimetric differences between pre-and post-plan but also lower the difficulty of puncture [6–8]. However, the number of samples included in the past studies was small, and the quality was uneven. So, we aimed to peform a systematic review and meta-analysis of related researches on the dosimetry after RIS implantation with or without template.

## Methods

### Study selection

The meta-analysis was carried out according to the PRISMA (Preferred Reporting Items for Systematic Reviews and Meta-Analyses) statement for reporting reviews and meta-analysis [9].

Major electronic literature databases were systematically searched, which included EMbase, PubMed, Cochrane Library, Wan Fang Med Online, CNKI. The search used various combination of subject words and free words, which included brachytherapy, iodine radioisotopes, iodine-125, 3-dimensional printing, 3D print. And the search strategy was determined after multiple presearches. Articles published before November 2020 were found in the search without publication and any language restriction. In addition, The researchers review the full texts of the included literature and carefully checked the list of references of the selected literature so as to avoid missing any other relevant researches on this topic.

### Inclusion criteria

In order to be included in this meta-analysis, the study had to meet all the criteria as follows: (1) randomized controlled trials (RCTs) or non-RCTs or retrospective study; (2) 2-arm studies in which patients received 3D print template-assisted RIS implantation in the treatment group and traditional free-hand implantation in the control group or a single-arm study reporting OAR with 3D print template-assisted RIS implantation; (3) Studies had outcomes of dosimetry or operation time.

### Exclusion criteria

The exclusion criteria include the following: (1) abstract, letter, case report, editorial, animal experiments, review, and other irrelevant studies; (2) no outcome measures.

### Data extraction

Two researchers (E.C. and Y.Z.) searched and reviewed related studies and carried out the data extraction independently. When there was any controversy, articles would be sent to a third reviewer (H.Z) for assessment until they achieved a agreement. We extract information for the following items: study characteristics (author, publication year, study design), demographic data (tumor site, sample size), treatment characteristics (with or without 3D print template), and outcome data including D90(the dose of 90% of the target volume), D100, V90(the percent of the tumor target receiving 90% of the prescribed dose), V100, V150, V200, D2cc (the dose received by 2 cm^3^ of normal tissue) and operation time.

### Quality assessment

For RCTs, the methodological quality were assessed by Cochrane risk of bias tool. Non-RCTs were assessed by the Newcastle–Ottawa Scale (NOS) [10]. Two researchers (E.C. and J.W.) carried out the scoring independently, and debated until a full agreement was reached. Studies with a score more than 7 were considered high quality, 4–6 moderate and below than 4 low quality.

### Statistical analysis

Standard mean difference (SMD) was adopted as the effect indicator for dosimetry and operation time. Pooled SMD and 95% confidence interval (CI) were calculated. We used *I*^2^ statistics to evaluate statistical heterogeneity. An *I*^2^ value of 0–40% indicates low heterogeneity; 30–60%, moderate heterogeneity; 50–90%, substantial heterogeneity; 75–100%, considerable heterogeneity. The Mantel–Haenszel fixed effect model [11] was applied for *p* > 0.1, *I*^2^ < 50%; data were pooled with the random-effects model when the *I*^2^ > 50%. *p* < 0.05 was considered to indicate that the difference was statistically significant. Publication bias was assessed with Egger’s regression. The statistical analysis were carried out using Stata 12.0 software.

## Results

### Literature search results

**Fig. 1:**
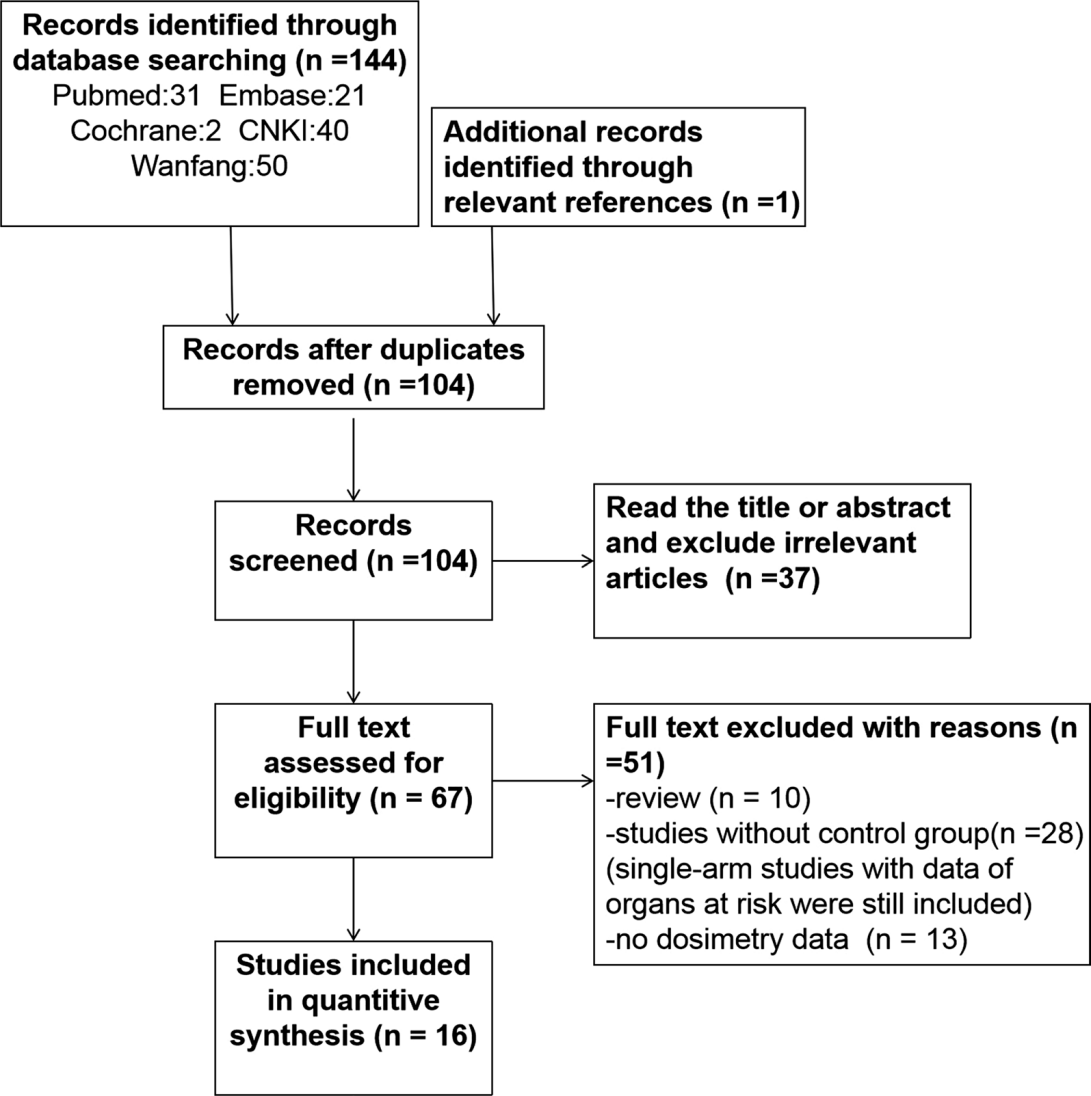
Flow diagram of the study selection process. *CNKI* China Knowledge Resource Integrated Database

In total, 145 candidate publications were retrieved. Ultimately, 16 studies [12–27] which fullfilled the eligibility criteria were included in the final analysis (see Fig. 1 for further details). All of the researches originated from China., including 16 non-RCTs. Eight original articles [12–19] with 280 patients compared the dose distribution differences between 3D print template-assisted implantation and traditional free-hand implantation. Among them, 128 patients received 3D print template-assisted RIS implantation and 152 without template. Five studies [20–24] with 76 patients compared the differences of D90, D100, V100, V150, V200, and D2cc of organs at risk (OAR) values pre- and post-implantation with 3D print template. In three studies [25–27], both 3DPNCT and 3DPCT plans were designed for all patients. The prescribed dose and seed activity were same. The data including D90, D100, V100, V150, V200, D2cc of OAR, number of seeds, number of needles and number of through bone needles in the two plans were compared. Basic information for inclusion in the study is presented in Table 1.

**Table 1:** Characteristics of the included studies

Ref	First author and year	Number of patients	Template	Tumor size		Prescription dose (Gy)		Tumor site	Outcomes	
				Template	Free hand	Template	Free hand		Dosimetric parameter	Operation time
1	Hongtao [12] 2016	13/14	3DPNCT	NA	NA	85.00 ± 33.10	84.43 ± 28.89	Multiple lesions	√	
2	Han [13] 2017	15/25	3DPNCT	≤ 3 cm: 14(lessions), 3–8 cm: 29, > 8 cm: 4	≤ 3 cm: 27, 3–8 cm:34, > 8 cm: 5	120	120	Liver	√	√
3	Huang [14] 2018	12/13	3DPCT	≤ 3 cm: 1, 3–5 cm:10, > 5 cm: 1	≤ 3 cm: 3, 3–5 cm:9, > 5 cm: 1	140	140	Pancreas	√	
4	Cao [15] 2017	10/10	3DPCT	NA	NA	120–160	120–160	Lung	√	
5	Pan [16] 2018	30/30	3DPNCT	64.4 ± 58.4(cc)	67.8 ± 60.4(cc)	119.46 ± 11.57	118.31 ± 11.41	Multiple lesions	√	√
6	Shen [17] 2018	28/32	3DPNCT	≤ 3 cm: 11, > 3 cm: 17	≤ 3 cm: 9, > 3 cm: 23	125.60 ± 23.60	123 ± 17.80	Head and neck	√	√
7	Zheng [18] 2019	13/10	3DPCT	25.9 ± 9.4	20.1 ± 5.3	90–120		Mediastinal lymph nodes	√	
8	Di [19] 2017	7/18	3DPNCT	≤ 5 cm: 13, > 5 cm: 12		60–100		Para-aortic lymph node	√	
9	Ji [20] 2017	21	3DPNCT	61.10 (4.0–263.0) cm ^3^		NA		Paravertebral/retroperitoneal Malignant Tumors	√	
10	Wang [21] 2016	10	3DPNCT	NA		NA		Pelvic recurrent rectal cancer	√	
11	Yuliang [22] 2016	15	3DPNCT	71.0 (7.0–167.3) cm ^3^		110–150 Gy		Pelvic recurrent cancer	√	
12	Jiang [23] 2017	9	3DPNCT	68.63 ± 62.83 cm ^3^		120 (110–160)Gy		Pelvic recurrent cervical cancer	√	
13	Zhe [24] 2017	21	3DPNCT	77.1(6.5–411.6)cm ^3^		150 (110–180)Gy		Chest malignant tumor	√	
14	Ji [25] 2019	33	Both	36.2(3.2–204.5)cm ^3^		160 (120–170)Gy		Peripheral lung cancer	√	
15	Ang [26] 2019	37	Both	40.0(4.6–332.4)cm ^3^		140 (100–180)Gy		Pelvic wall recurrent gynecological Malignant tumors	√	
16	Xuemin [27] 2018	10	Both	NA		NA		Superficial sarcoma	√	

3DPNCT: 3D print noncoplanar template; 3DPCT: 3D print coplanar template;√: the data is available

### Quality evaluation

All included studies were assessed using Newcastle Ottawa Scale. All 2-arm studies [12–19] achieved a score of ≥ 7. Five studies [20–24] comparing dosimetry values pre- and post-implantation with 3D print template achieved a score of 6. Three studies [25–27] comparing 3DPNCTwith 3DPCT plans achieved a score of 9 (as shown in Table 2).

**Table 2:** Quality assessment of included studies

First author and year	Representativeness^a^	Selection of non-exposed ^b^	Ascertainment of exposure^c^	Incident disease^d^	Comparability^e^	Assessment of outcome^f^	Length of follow-up^g^	Adequacy of follow-up^h^
Hongtao [12] 2016	A	A	A	A	C	B	A	A
Han [13] 2017	A	A	A	A	B	B	A	A
Huang [14] 2018	A	A	A	A	B	B	A	A
Cao [15] 2017	A	A	A	A	C	B	A	A
Pan [16] 2018	A	A	A	A	B	B	A	A
Shen [17] 2018	A	A	A	A	A	B	A	A
Zheng [18] 2019	A	A	A	A	C	B	A	A
Di [19] 2017	A	A	A	A	C	B	A	A
Ji [20] 2017	A	N.A	A	A	N.A	B	A	A
Wang [21] 2016	A	N.A	A	A	N.A	B	A	A
Yuliang[22] 2016	A	N.A	A	A	N.A	B	A	A
Jiang [23] 2017	A	N.A	A	A	N.A	B	A	A
Zhe [24] 2017	A	N.A	A	A	N.A	B	A	A
Ji [25] 2019	A	A	A	A	A	B	A	A
Ang [26] 2019	A	A	A	A	A	B	A	A
Xuemin [27] 2018	A	A	A	A	A	B	A	A

^a^A truly representative, B somewhat representative, C selected group, D no description of the derivation of the cohort

^b^A drawn from the same community as the exposed, B drawn from a different source, C no description of the derivation of the non-exposed

^c^A secure record, B structured interview, C written self-report, D no description

^d^Demonstration that the outcome of interest was not present at start of study: A yes, B no

^e^A study controls for demographics/comorbidities, B study controls for any additional factor (e.g., age, severity of illness), C not done

^f^A independent or blind assessment, B record linkage, C self-report, D no description

^g^Long enough for outcomes to occur? A yes, B no

^h^A complete follow-up, B subjects lost to follow-up was unlikely to introduce bias, C follow-up rate 90% or lower, D no statement

### Dosimetry

**Fig. 2:**
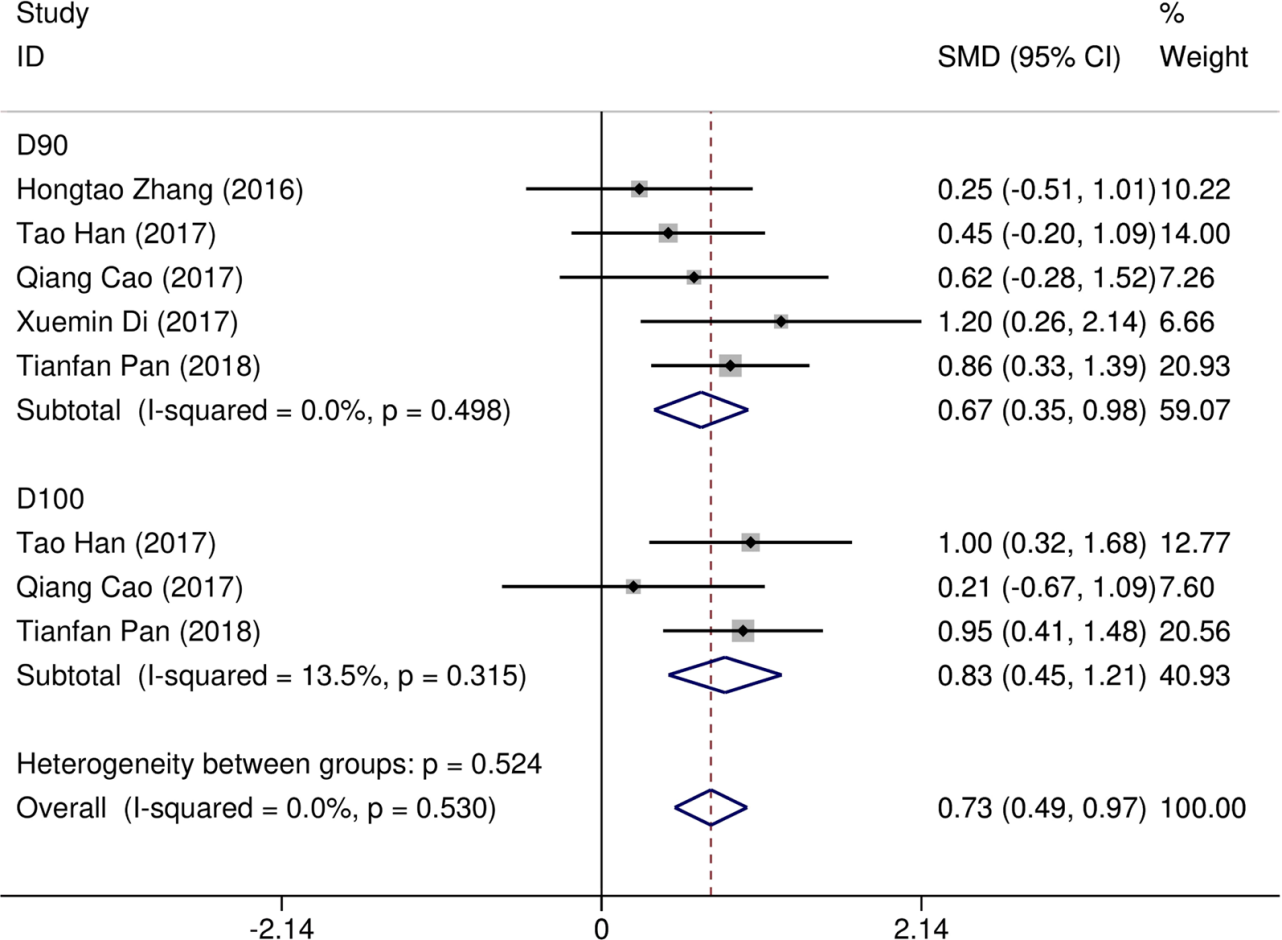
Forest plot of studies comparing post-implantation D90 and D100 between 3D print template and traditional free-hand implantation

**Fig. 3:**
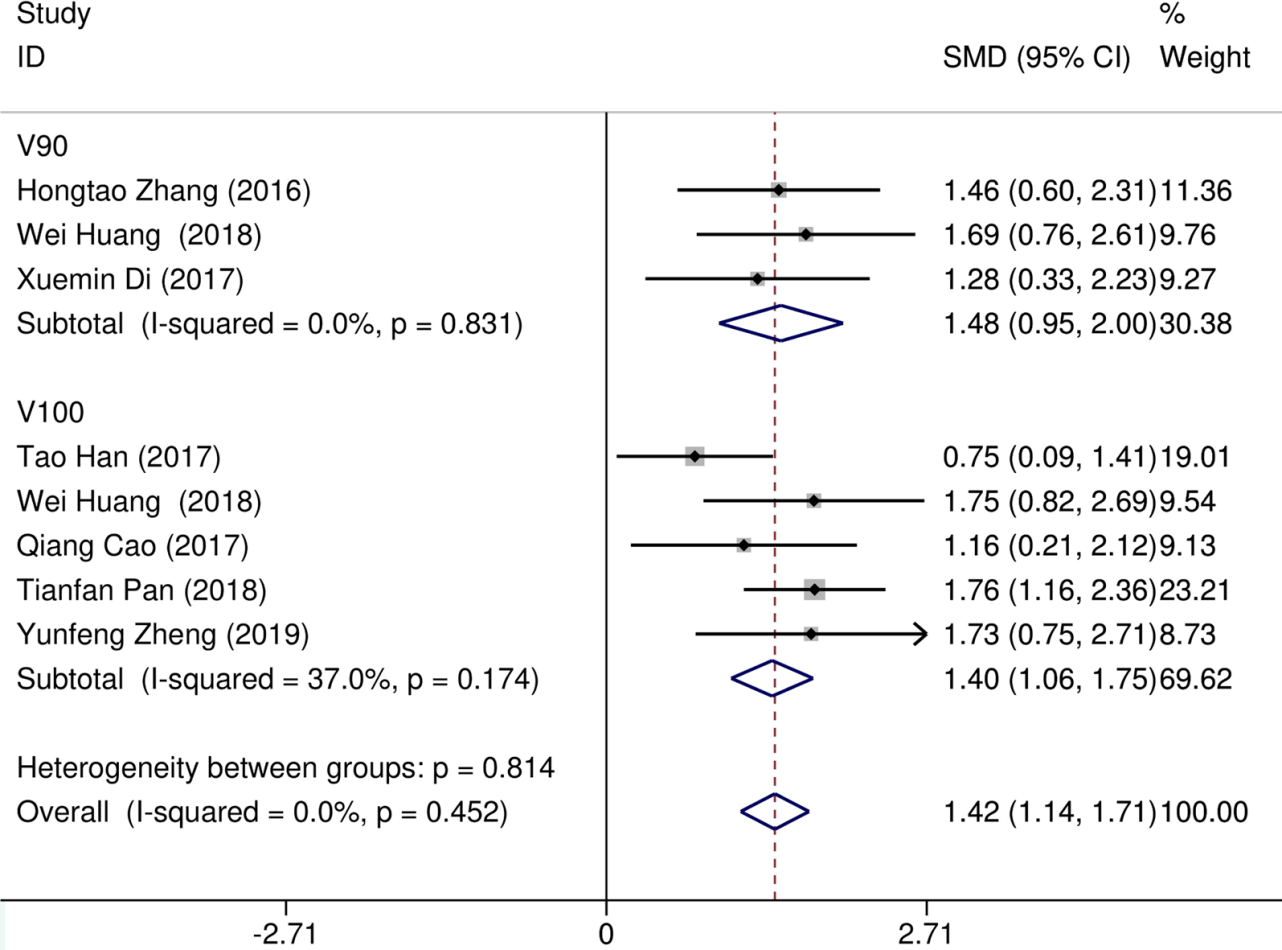
Forest plot of studies comparing post-implantation V90 and V100 between 3D print template and traditional free-hand implantation

**Fig. 4:**
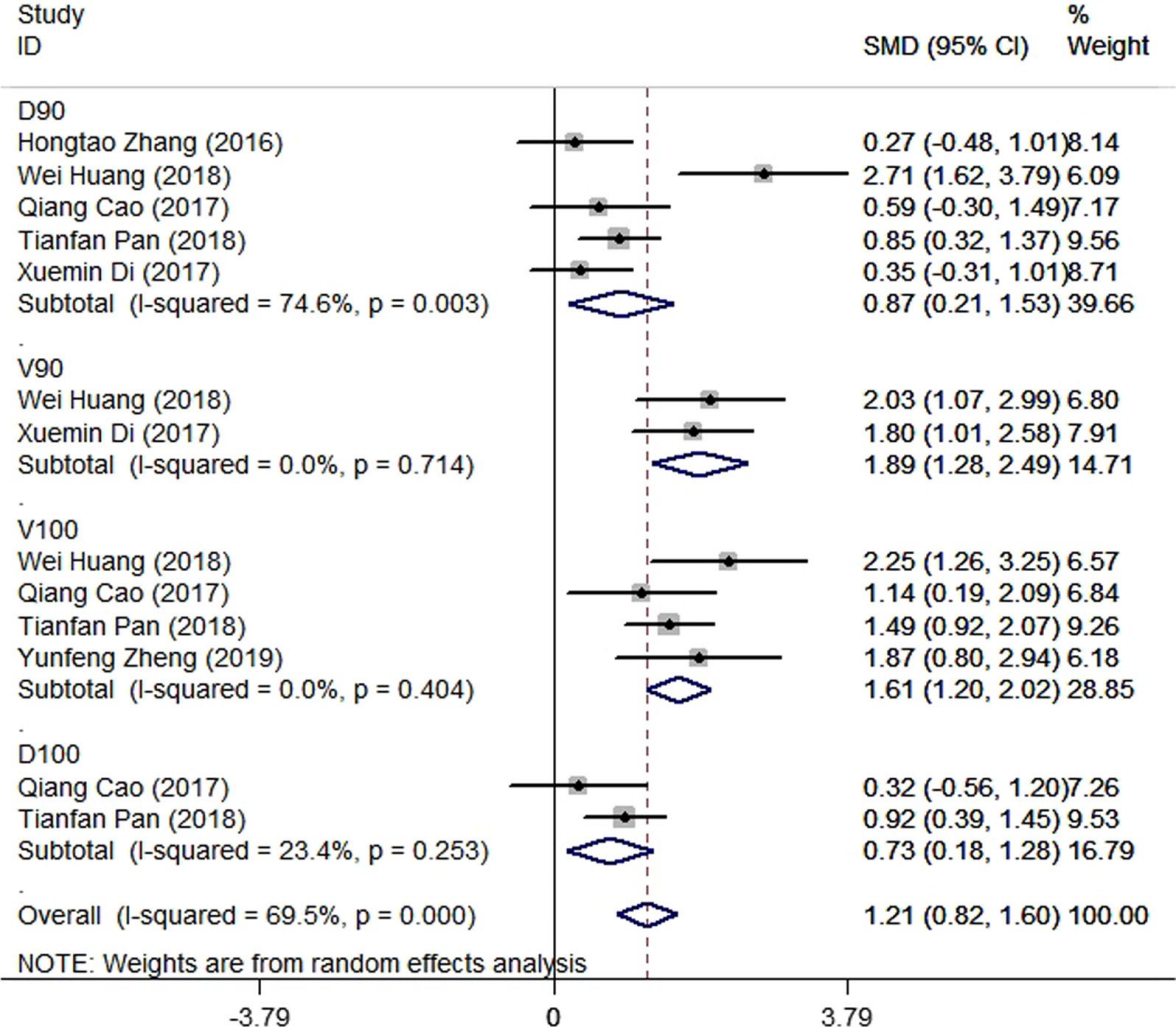
Forest plot of studies comparing D90, D100, V90, and V100 between pre- and post-implantation with traditional free-hand implantation

**Fig. 5:**
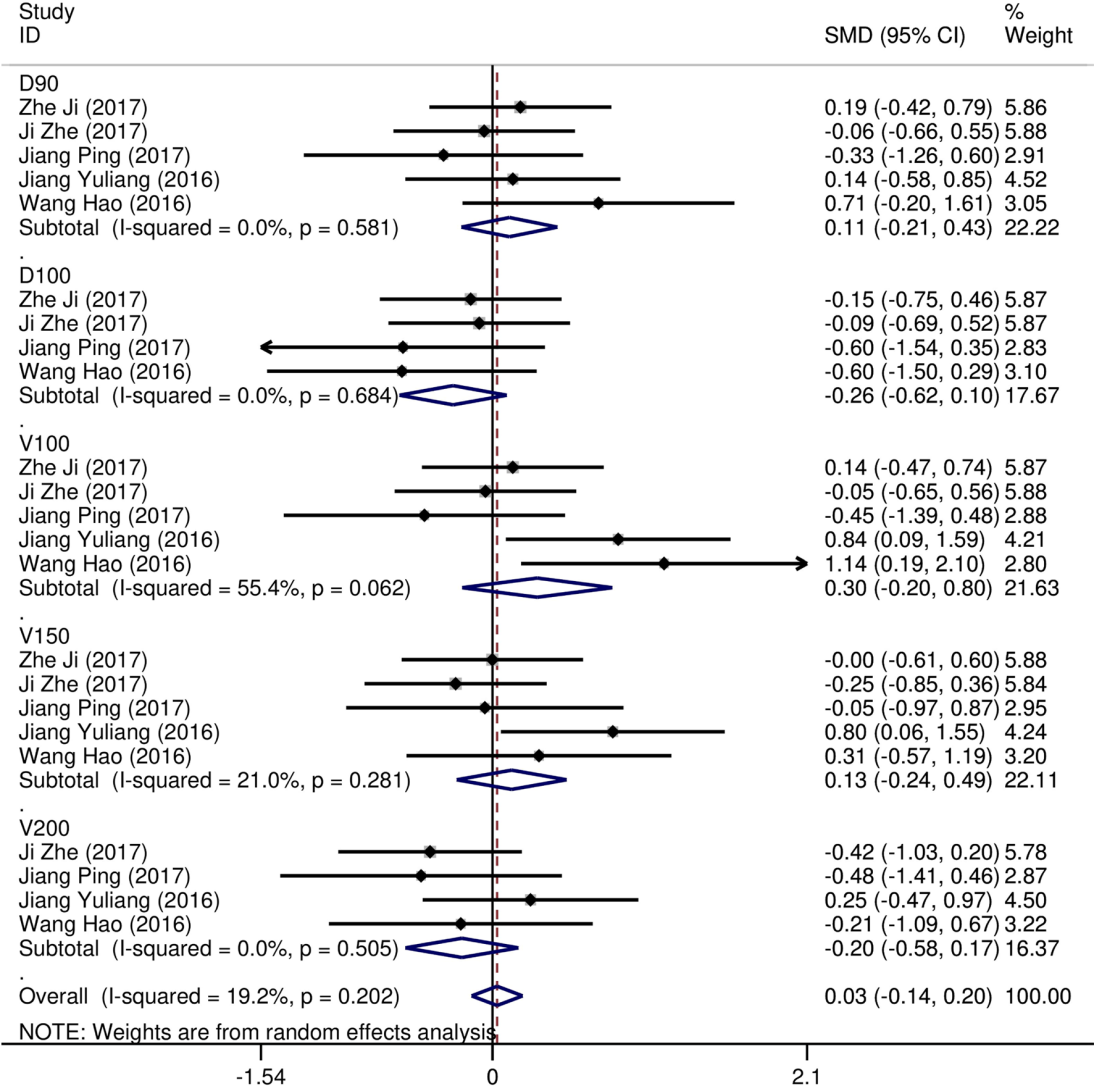
Forest plot of studies comparing D90, D100, V100, V150, and V200 between pre- and post-implantation with 3D print template

**Fig. 6:**
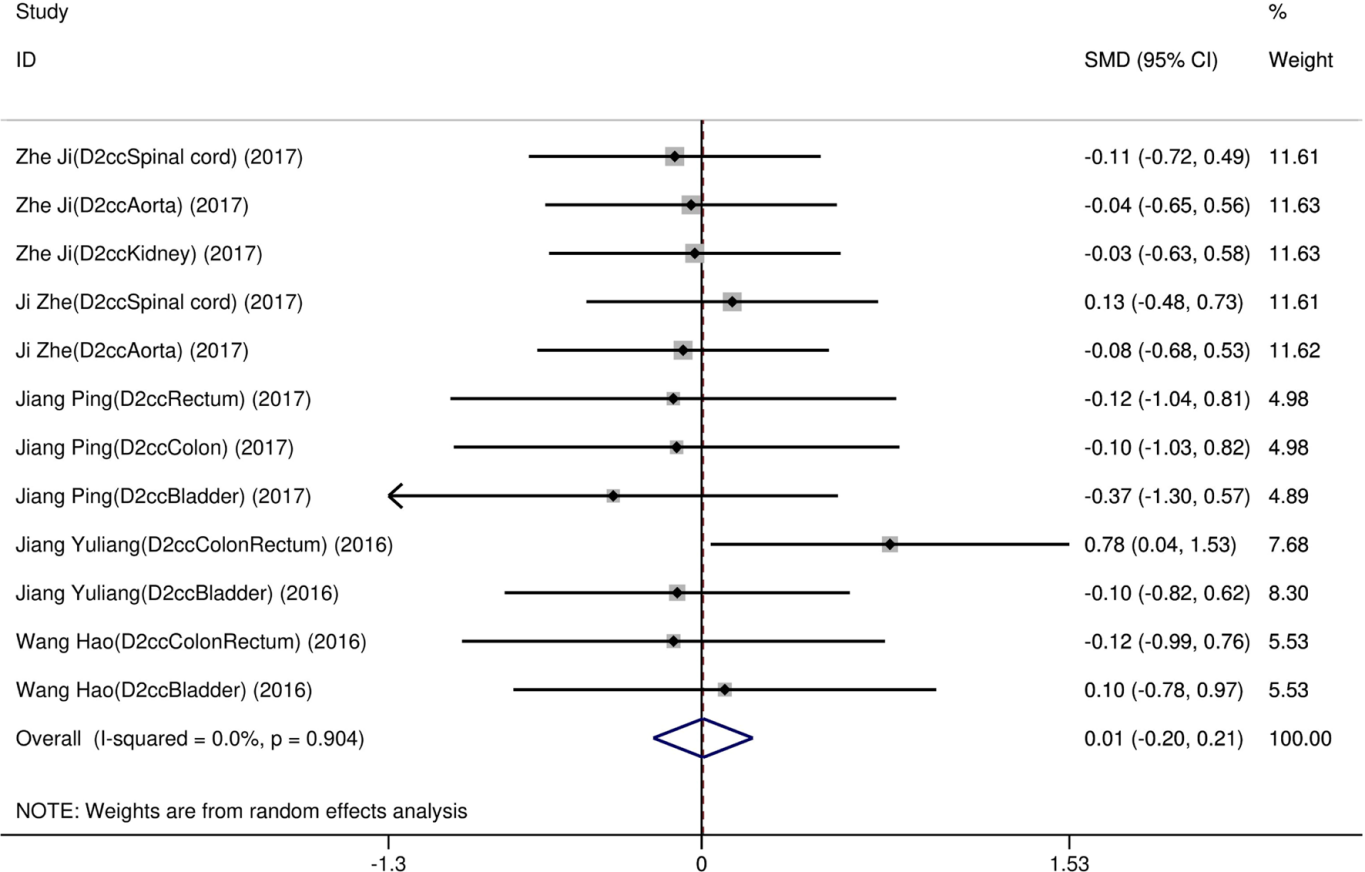
Forest plot of studies comparing D2cc of OARs between pre- and post-implantation with 3D print template

**Fig. 7:**
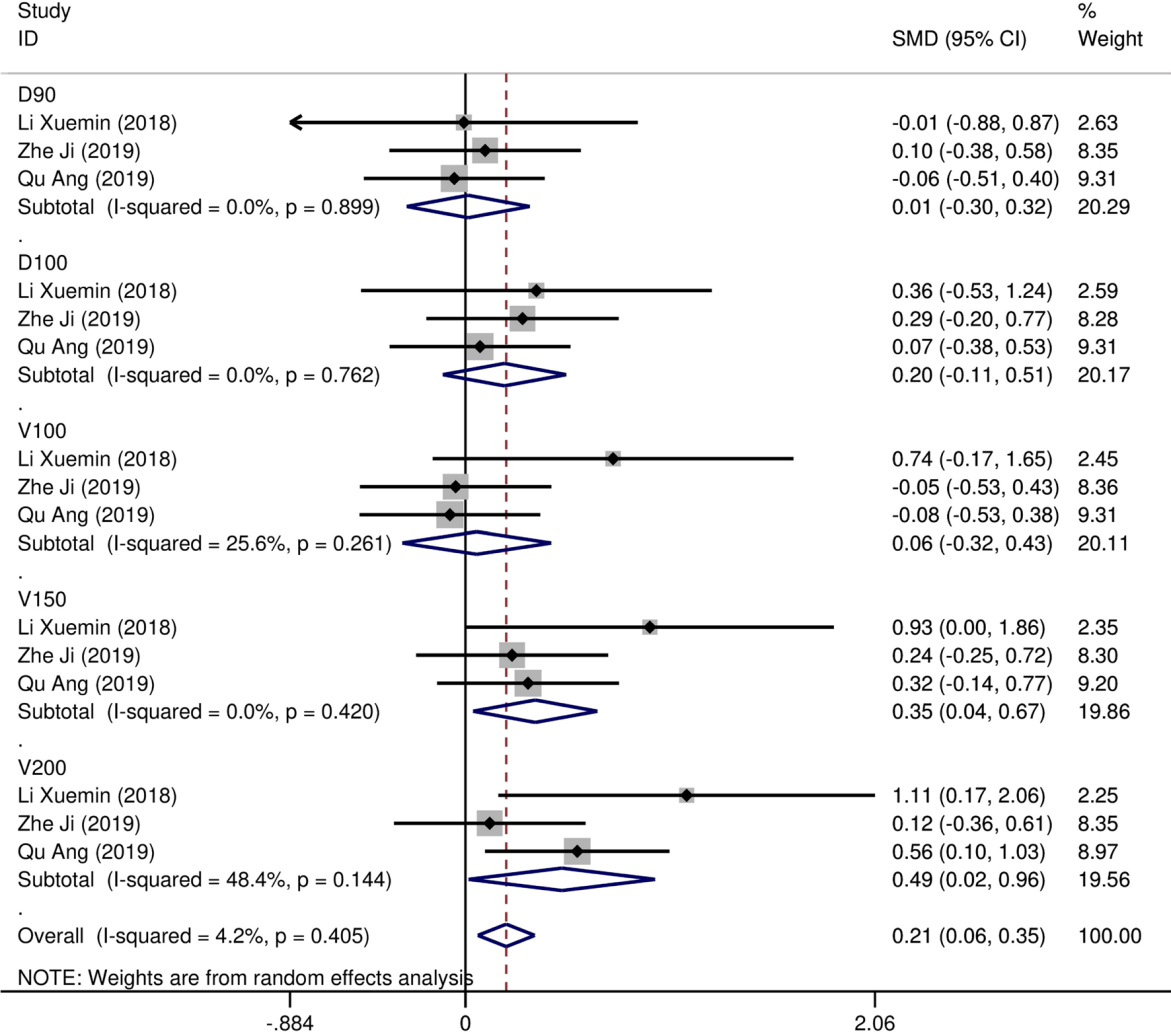
Forest plot of studies comparing D90, D100, V100, V150 and V200 between 3DPNCT and 3DPCT groups

**Fig. 8:**
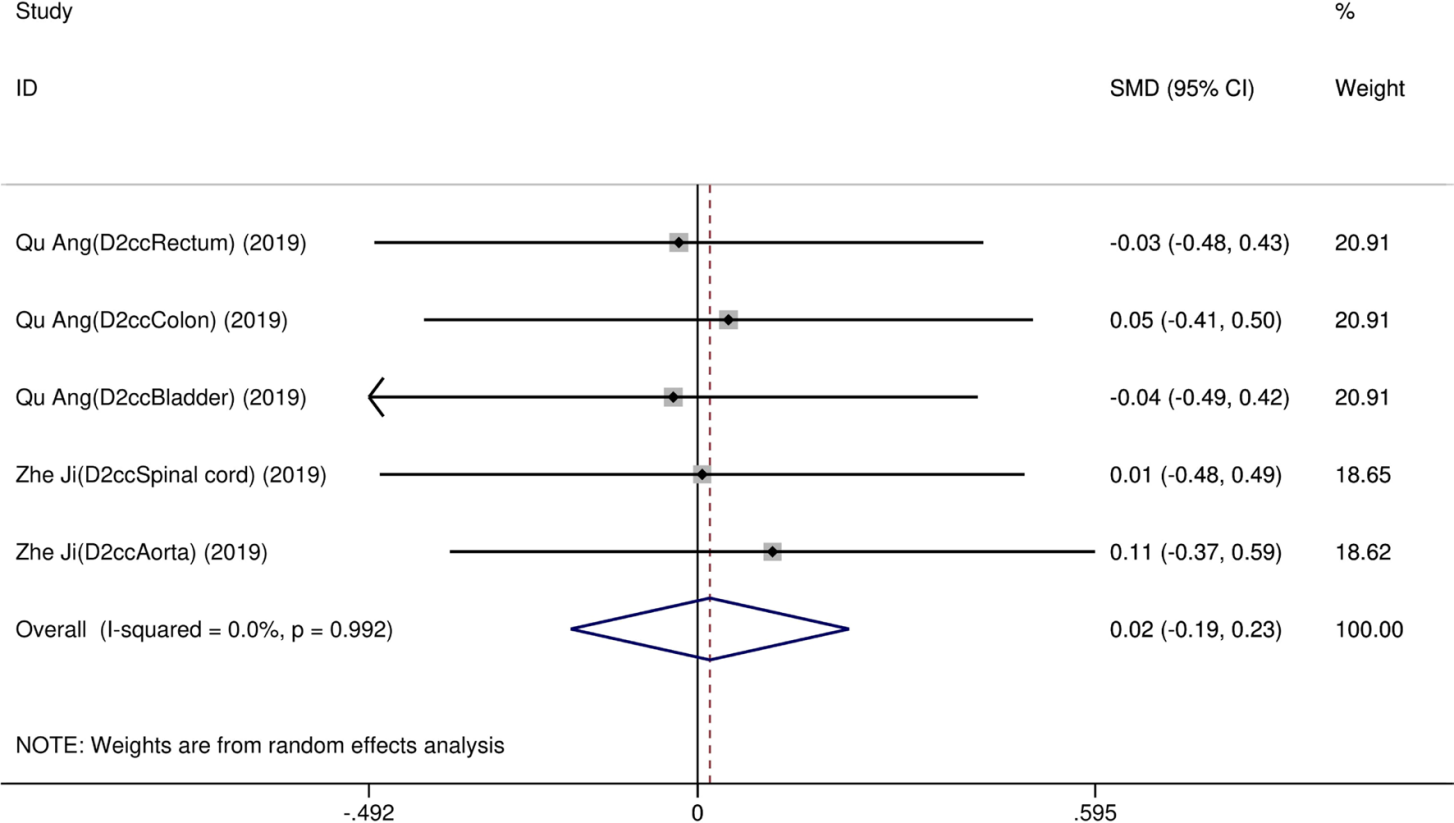
Forest plot of studies comparing D2cc of OAR between 3DPNCT and 3DPCT groups

**Fig. 9:**
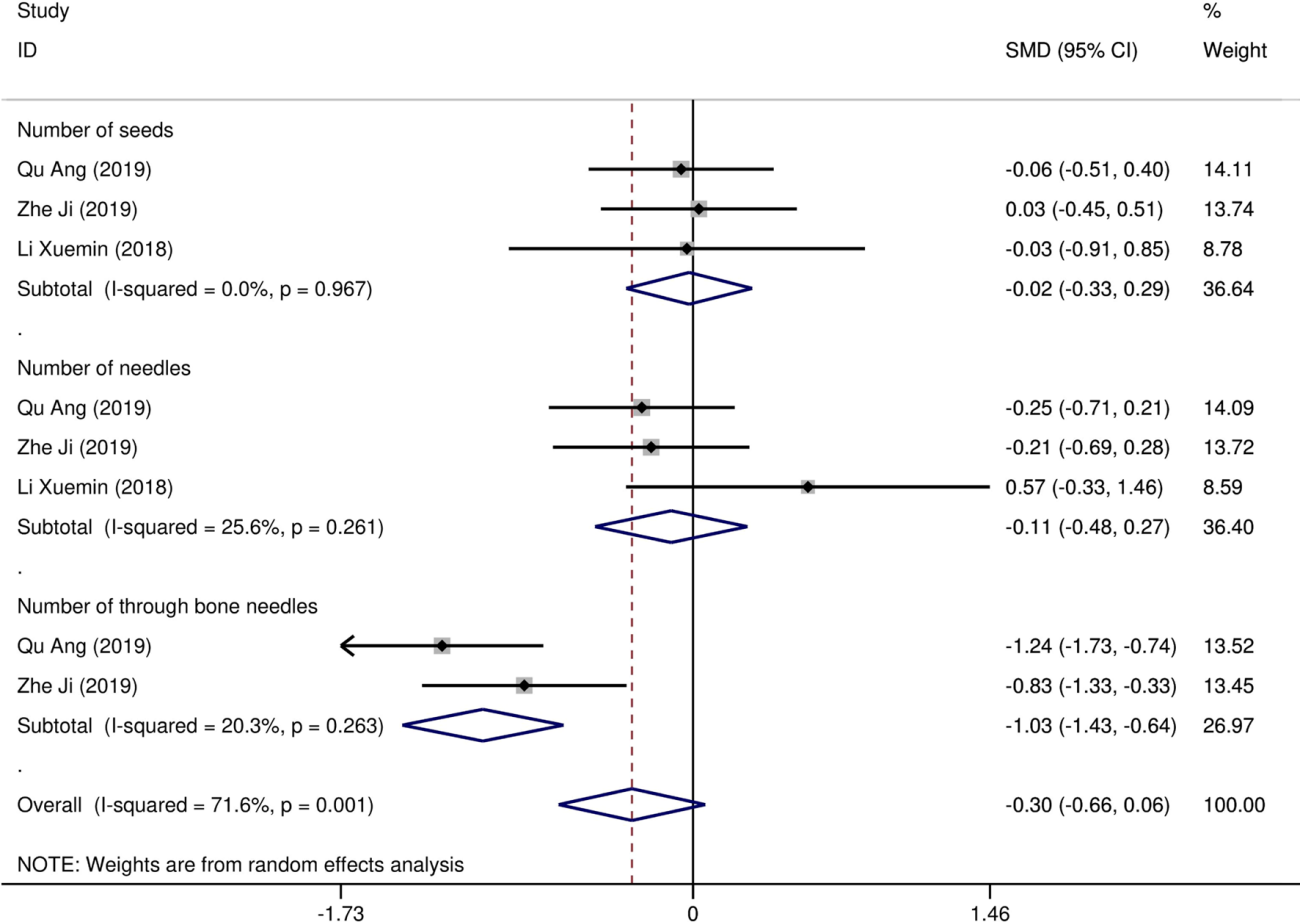
Forest plot of studies comparing number of seeds, number of needles and through bone needles between 3DPNCT and 3DPCT groups

Eight studies [12–19] compared post-implantation dosimetry data between 3D print template and traditional free-hand implantation. For D90, D100, V90, V100, there occurred no significant heterogeneities among results (*I*^2^ = 0, 0, 0, and 37%, respectively; *p* = 0.498, 0.315, 0.831, and 0.174, respectively). The pooled effect was therefore evaluated using a fixed-effects model. The result showed that all of these dosimetric parameters in 3D print template group were higher than those in traditional group with statistically significant (SMD = 0.67, 0.82, 1.48, and 1.41, respectively; 95%CI = 0.35 to 0.98, 0.40 to 1.23, 0.95 to 2.00, 0.96 to 1.86, respectively; *p* < 0.001) (as shown in Figs. 2 and 3). Six studies [12, 14–16, 18, 19] reported dosimetry pre- and post-implantation with traditional free-hand implantation. The result showed that all dosimetric parameters including D90, D100, V90, and V100 values showed significant differences between pre- and post-implantation (SMD = 0.87, 0.73, 1.89, and 1.61, respectively; 95%CI = 0.21 to 1.53, 0.18 to 1.28, 1.28 to 2.49, and 1.20 to 2.02 respectively; *p* = 0.010, 0.010, < 0.001, and < 0.001, respectively)(as shown in Fig. 4). Five studies [20–24] reported dosimetry pre- and post-implantation with 3DPNCT. The result showed that all dosimetric parameters including D90, D100, V100, V150, V200 and D2cc of OARs showed no significant differences between pre- and post-implantation(SMD = 0.11, − 0.26, 0.30, 0.13, − 0.20, and 0.01, respectively; 95%CI = − 0.21 to 0.43, − 0.62 to 0.10, − 0.20 to 0.80, − 0.24 to 0.49, − 0.58 to 0.17, and − 0.20 to 0.21 respectively; *p* = 0.489, 0.151, 0.243, 0.494, 0.289, and 0.954, respectively) (as shown in Figs. 5 and 6). In three studies [25–27], both 3DPNCT and 3DPCT plans were designed for all patients. Pooled analysis of D90, D100, V100, D2cc of OAR, number of seeds and number of needles showed no significant differences between 3DPNCT and 3DPCT groups (*p* = 0.930, 0.215, 0.766, 0.863, 0.904, and 0.575, respectively). V150, V200 increased (SMD = 0.35, 0.49; 95%CI = 0.04 to 0.67, 0.02 to 0.96; *p* = 0.028, 0.043) and number of through bone needles decreased (SMD = − 1.03, 95%CI = − 1.43 to − 0.64, *p* < 0.001) with 3DPNCT (as shown in Figs. 7, 8 and 9).

### Operation time

Three studies reported operation time. The heterogeneity result showed no significant heterogeneity (*I*^2^ = 35.4%, *p* = 0.213). The fixed effect model was utilized. The result showed that 3D print template assisted RIS implantation could reduce operation time with statistically significant compared to taditional free-hand implantation (SMD = − 0.93; 95%CI = − 1.3 to − 0.51; *p* < 0.001) (as shown in Additional file 1: Fig. 1).

### Publication bias

For studies comparing 3D print template-assisted implantation with traditional free-hand implantation, publication bias was evaluated by a funnel plot. The regression test of the funnel plot symmetry confirmed that no publication bias was found (*p* = 0.999, 0.373, 0.903, 0.833, and 0.964, respectively) for D90, D100, V90, V100, and operation time (as shown in Additional file 2: Fig. 2, Additional file 3: Fig. 3).

## Discussion

RIS implantation has the dosimetric advantage of sharp dose gradients between tumor target area and adjacent normal tissues, which allows more sparing of the surrounding organs at risk. It has been widely used in China for the following tumors: head and neck, thorax, breast, abdomen, and pelvic cavity; and it has a good effect on relieving pain, reducing tumor burden, improving life quality and prolonging survival time of patients [28–32]. But except for prostate cancer, there is still no standard method to treat other tumors. In the past, ^125^I seeds were implanted just by doctors’ experience. However, it is challenging for doctors to insert many needles at 1 time into the targer in line with the preplan, which leads to a large difference in location of seeds and dose distribution between pre- and post- plan, finally leading to tumor local recurrence and complications [12–19]. Also, traditional free-hand ^125^I seed implantation is very complicated and time consuming; the operators usually spend a lot of time learning these special skills [16, 17]. Therefore, how to achieve a precise distribution of RIS in the tumor target area and to ensure that RIS implantation treatment is strictly followed up as preplanning are the research focuses.

3D print template is a personalized template which contains preset implant channel information and body surface information of patients' treatment area. Through accurate intraoperative reset of template and real-time planning of TPS, the consistency between actual implant channel and planned needle channel can be improved. With the assistence of a 3D print template, it is easy and efficient to insert needles at any arbitrary angle into the tumor target, meanwhile accurately reproducing the needle positions according to the preplan. This method has been shown to improve the accuracy of seed location and dose distribution in many studies [9–13], but as far as we know, there is still a lack of evidence-based medical data regarding its effectiveness.

Our meta-analysis result firstly showed that there was no significant difference between pre- and post-implantation for all the parameters including D90, D100, V100, V150, V200 and D2cc of OARs with 3D print template and that the D90, D100, V90 and V100 in the template group were higher than those in the freehand group, indicating that 3D print template could provide good accuracy for RIS implantation. And through the template guidance, the operation time was also reduced. Liang et al. [7] treated 15 patients with cervical lymph node metastasis by 3D print template assisted RIS implantation, and found that the dose distribution in preplan can be achieved easily and satisfactorily by 3D print template. Zhang et al. [8] used 3D print template to assist RIS implantation in 14 patients, and the differences of D90, V90, V100 and V150 values pre- and post-implantation showed no statistically significant. Zhang et al. [12] analyzed the clinical data of 27 patients with RIS implantation (13 patients with template, 14 with freehand); the result showed that V90 (92.76% ± 1.89%) in the template group was significantly higher than that in the freehand group (84.59% ± 7.56%), the difference was statistically significant (*p* = 0.001). Huang et al. [33] treated 25 patients with head and neck tumors by RIS implantation with the guidance of 3D print template. According to the insertion site of the needle, the patients were divided into four groups: parotid gland and masseter area group (9 cases); maxillary and paranasal area group (8 cases); submandibular and upper neck area group (5 cases); posterior area group (6 cases). All the needles were inserted at their predetermined positions once. The average insertion time was 7.5 s for each needle, and no complications were observed. These studies all have indicated that 3D print template could not only improve postoperative dose distribution but also lower the difficulty of puncture and reduce the operation time.

In this meta-analysis, we also found that both 3DPNCT and 3DPCT plans could achieve prescription dose. Pooled analysis of D90, D100, V100, and D2cc of OAR showed no significant differences between 3DPNCT and 3DPCT groups. But compared with 3DPCT, in 3DPNCT group, both V150 and V200 were increased, indicating that 3DPNCT could increase the volume of high dose within tumor target. And the larger volume of high dose might produce more beneficial effects on local control. On the other hand, the number of through bone needles was reduced in 3DPNCT group, which showed that 3DPNCT was more safer in the respect of puncture route. However, there are several challenges in the broader use of 3D print template, which include good preoperative implantation designing, accurate 3D print template calibration, and the confidence of doctors in carrying out the procedures. The template is advantageous in challenging clinical cases, which include tumors close to important organs and tissues, blocked by the bones, or next to the important blood vessels. On the other hand, it is time-consuming to design and prepare 3D print template, though the needle could be inserted into the target more accurately and efficiently, leading to better dose consistency.

In this study, the advantages of 3D print template in RIS implantation were revealed by evidence-based medicine study for the first time. Our meta-analysis result showed that both 3DPNCT and 3DPCT assisted RIS implantation can realize the accurate distribution of RIS, and make the post implantation dosimetry more predictable, which provided evidences for clinical practice. However, there are shortcomings in our study: as the relevant studies included were all from China, it may lead to the selection bias of the literature. Also, we used SMD as the effect indicator for dosimetry and operation time to perform meta-analysis due to the differences in preplan among studies. The advantage of 3D print template in clinical efficacy such as local tumor control and long-term survival of patients still needs a larger sample and high-quality randomized controlled trial to verify in the future.

## Conclusion

Our meta-analysis result showed that 3D print template assisted RIS implantation can realize the accurate distribution of RIS, optimize dose distribution and reduce the operation time at the same time. Compared with 3D print coplanar template, 3D print noncoplanar template could increase the volume of high dose within tumor target and was more safer in the respect of puncture route, which provided evidences for clinical practice.

## Supplementary Information

**Additional file 1**. Forest plot of studies evaluating operation time.

**Additional file 2**. Funnel plot of studies evaluating D90, D100.

**Additional file 3**. Funnel plot of studies evaluating V90, V100.

## Data Availability

The datasets analysed for this study are available from J.W. on reasonable request.

## Abbreviations

3D: Three-dimensional
3DPNCT: 3D print noncoplanar template
3DPCT: 3D print coplanar template
CNKI: China National Knowledge Infrastructure
CI: Confidence interval
SMD: Standard mean difference
RIS: Radioactive iodine-125 seeds
PRISMA: Preferred Reporting Items for Systematic Reviews and Meta-Analyses
RCTs: Randomized controlled trials
D90: The dose of 90% of the target volume
D100: The dose of 10% of the target volume
V90: The percent of the tumor target receiving 90% of the prescribed dose
V100: The percent of the tumor target receiving 100% of the prescribed dose
V150: The percent of the tumor target receiving150% of the prescribed dose
V200: The percent of the tumor target receiving 200% of the prescribed dose
D2cc: The dose received by 2 cm^3^ of normal tissue
