# Supplementary figures and images for: Dosimetry study of three-dimensional print template for 125I implantation therapy
Source: Radiat Oncol. 2021 Jun 24;16:115. doi: 10.1186/s13014-021-01845-y (PMC8223396; doi:10.1186/s13014-021-01845-y)

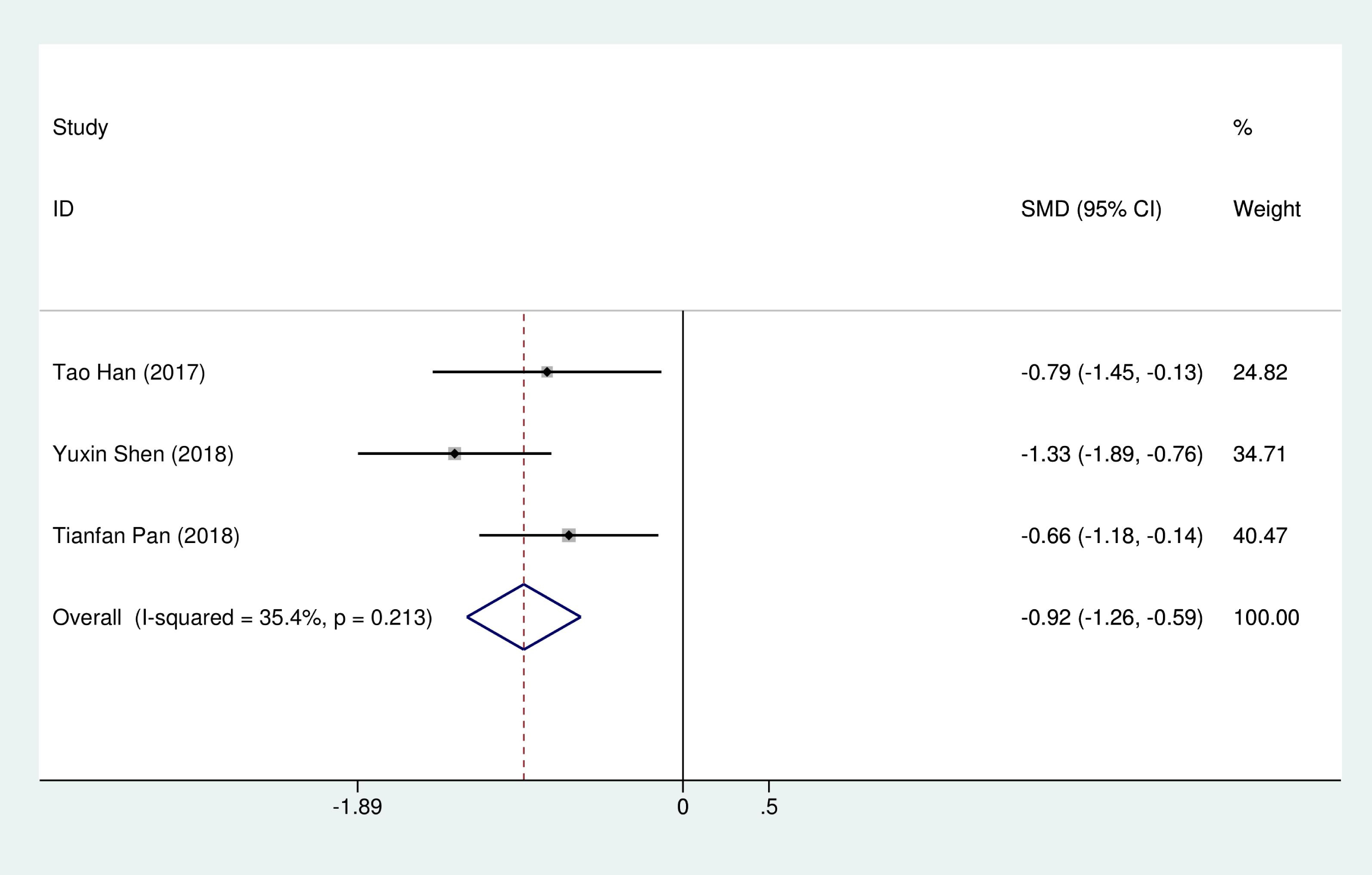

Supplement: Supplementary file 1 — Additional file 1. Forest plot of studies evaluating operation time. [file 13014_2021_1845_MOESM1_ESM.jpg]

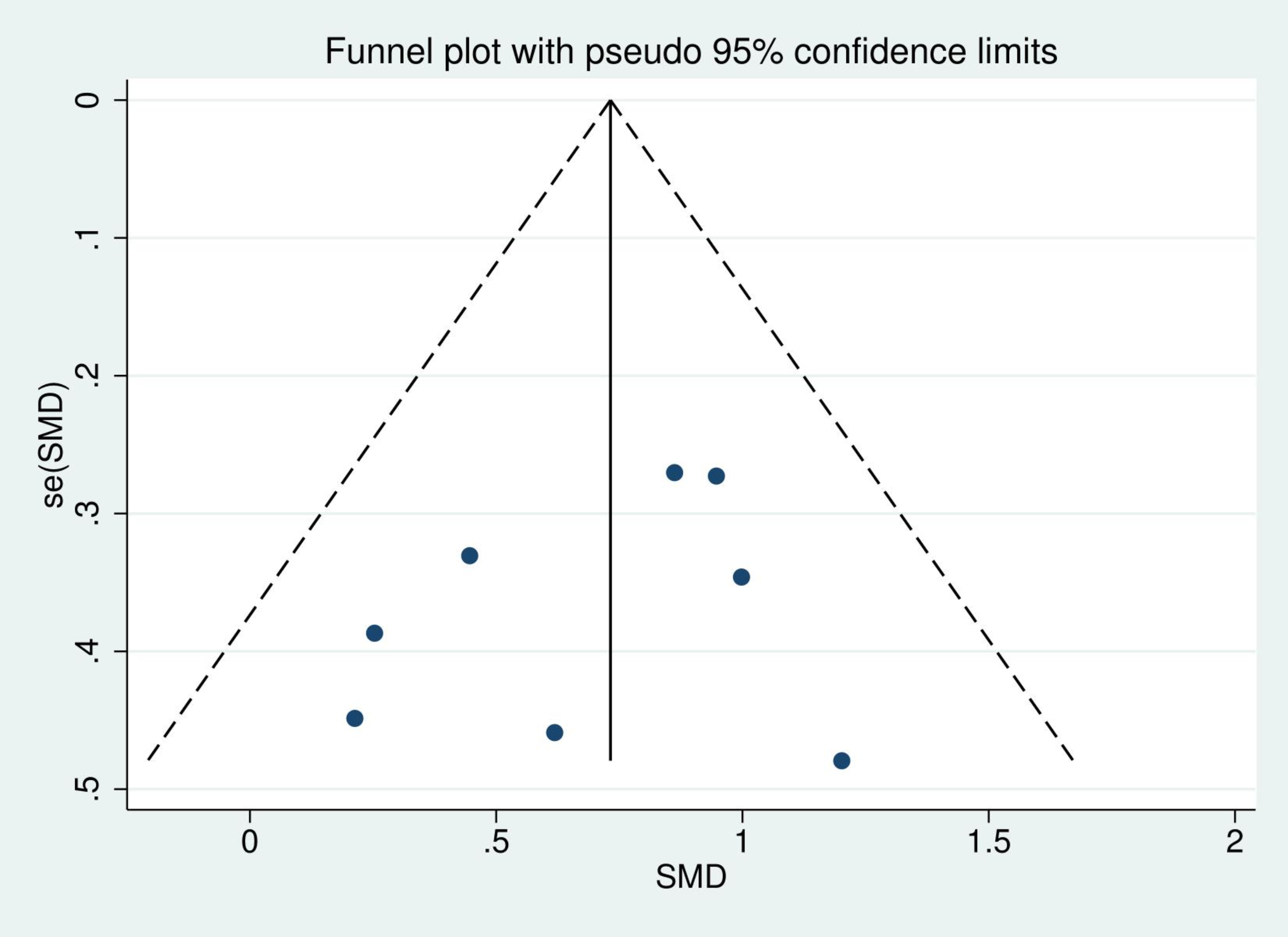

Supplement: Supplementary file 2 — Additional file 2. Funnel plot of studies evaluating D90, D100. [file 13014_2021_1845_MOESM2_ESM.jpg]

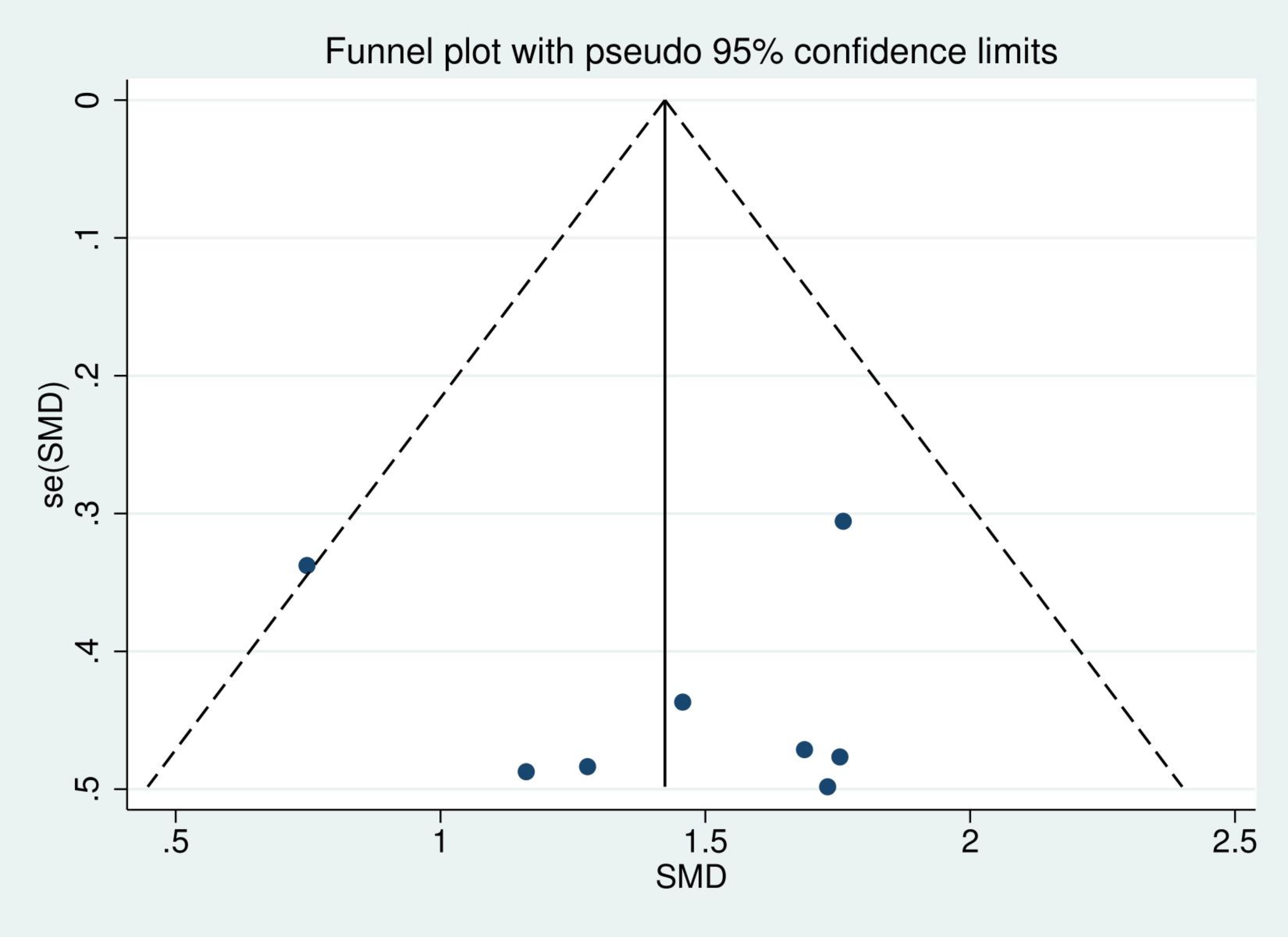

Supplement: Supplementary file 3 — Additional file 3. Funnel plot of studies evaluating V90, V100. [file 13014_2021_1845_MOESM3_ESM.jpg]
